# Treatment of As(III)-Laden Contaminated Water Using Iron-Coated Carbon Fiber

**DOI:** 10.3390/ma15124365

**Published:** 2022-06-20

**Authors:** Dun Fu, Tonni Agustiono Kurniawan, Herong Gui, Songbao Feng, Qian Li, Mohd Hafiz Dzarfan Othman

**Affiliations:** 1 Key Laboratory of Mine Water Resource Utilization of Anhui Higher Education Institutes, School of Resources and Civil Engineering, Suzhou University, Suzhou 234000, China; heronggui@ahszu.edu.cn (H.G.); songbaofeng@ahszu.edu.cn (S.F.); qianli@ahszu.edu.cn (Q.L.); 2 Key Laboratory of Estuarine Ecological Security and Environmental Health, Tan Kah Kee College, Xiamen University, Zhangzhou 363105, China; 3 College of the Environment and Ecology, Xiamen University, Xiamen 361102, China; 4 Advanced Membrane Technology Research Centre (AMTEC), School of Chemical and Energy Engineering, Universiti Teknologi Malaysia, Skudai 81310, Johor, Malaysia; hafiz@petroleum.utm.my

**Keywords:** adsorption, arsenic, carbon fiber, phyico-chemical technique, wastewater treatment

## Abstract

This work presents the fabrication, characterization, and application of iron-coated carbon fiber (Fe@CF), synthesized in a facile in situ iron reduction, for As(III) removal from an aqueous solution. The physico-chemical properties of the composite were characterized using Brunauer–Emmett–Teller (BET) surface area, scanning electron microscopy (SEM), X-ray diffraction (XRD), and Fourier-transform infrared (FTIR) spectroscopy. Adsorption studies were evaluated in batch experiments with respect to reaction time, the dose of adsorbent, As(III) initial concentration, pH, and co-existing ions. The results showed that the BET surface area and pore volume of Fe@CF slightly decreased after Fe coating, while its pore size remained, while the SEM and XRD analyses demonstrated that the Fe was successfully anchored on the CF. A maximum As(III) adsorption of 95% was achieved with an initial As concentration of 1.5 mg/L at optimum conditions (30 min of reaction time, 1 g/L of dose, 1 mg/L of As(III) concentration, and pH 3.5). Since the treated effluents could not meet the strict discharge standard of ≤10 μg/L set by the World Health Organization (WHO), a longer reaction time is required to complete the removal of remaining As(III) in the wastewater effluents. As compared to the other adsorbents reported previously, the Fe@CF composite has the highest As(III) removal. Overall, the findings suggested that the use of Fe@CF as an adsorbent is promising for effective remediation in the aquatic environment.

## 1. Introduction

As the result of water pollution that has threatened over 2 billion people recently, clean water has become an important issue in terms of pollution abatement and water recycling. To maintain a green environment for future generations, a clean water supply is vital due to its key role in addressing the world’s challenges such as food insecurity.

While clean water is important for public health, the extent of water pollution has risen in the developing world such as India due to arsenic (As) contamination that causes skin cancer or keratosis. There is no break, even during this Covid-19 global pandemic, as 300 million people are still affected by the As contamination [1]. The increasingly stringent discharge limits of arsenic (<10 μg/L) set by the WHO also result in the increasing demand for clean water [2]. Unless properly addressed, it is anticipated that by 2030, half of the world’s population will be living in water-stressed areas [1]. As everyone has the right to safe, clean, and affordable water for personal and domestic utilization, finding sustainable solutions for this problem represents another challenge for the current generation.

To address this global challenge, water scientists have constantly searched for new technologies that could be implemented in wastewater treatment operations. Various water technologies have been tested such as membrane filtrations [3], precipitation [4], and advanced oxidation process [5]. On the basis of economical, eco-friendly, and treatment performance, adsorption represents one of the most promising options to remove As from contaminated wastewater [6]. Adsorbents from low-cost sources such as agricultural waste [7], industrial by-products [8], and natural minerals [9,10] have been investigated for the treatment of contaminated water laden with heavy metals such as As, Cr, and Ni.

However, most of the adsorbents have not been investigated with respect to sustainability [11]. The sustainability approach of adsorption is based on the adsorbent with high surface area, ease of separation post-treatment, and structural and functional activity even after regeneration [12,13]. With such characteristics, identifying suitable functional materials that decrease the consumption of natural resources has become an imperative task for water scientists to contribute to the UN SDGs #6 “Clean water and sanitation” [14,15].

In this regard, new materials are more than often standing at the basis of technological breakthroughs, while water chemistry is enabling science to make game-changing solutions possible for an efficient sewage treatment [14]. Materials from unused resources can play roles when it comes to promoting a circular economy (CE). Adopting resource recovery and zero-waste approaches not only minimizes waste generation by utilizing by-products, but also paves the way forward for a closed loop in the CE [15]. As a green approach to water technology is the need of the hour, the use of functional materials for the removal of aquatic pollutants has intensified recently. Since the world’s economy gradually shifts toward a CE [16], there is increasing pressure to substitute conventional materials with sustainable and renewable materials such as composites from unused resources, which often end up in landfill for disposal [17].

Recently, combining two starting materials into a composite has gained popularity [18]. Although they are ubiquitous, the utilization of composites for water treatment is still limited [19]. If properly recycled and reused in the loop of a CE, the materials can lead to greenhouse gas emissions (GHG) reduction and carbon footprint attenuation [20]. To offer readers a new perspective of CE applications in water treatment, the application of composites as adsorbents could help public water utilities to attain carbon-neutral water treatment by providing treated water without generating secondary waste. This distinguishes its utilization as an adsorbent from other separation technology that still generates waste such as sludge that needs to be treated first before its final disposal in landfills. As a result, this adds operational costs to wastewater treatment.

To mitigate the bottlenecks in the field of study, iron-based composites have been developed to remediate As(III)-laden water because of their ability to disperse Fe nanoparticles. Among the composites, carbon fibers, made from polyacrylonitrile, were selected for this study due to their large surface area. As iron-coated carbon fiber (CF) exhibits favorable adsorption towards contaminants such as Cr(VI) [21,22], it may be regenerated for subsequent treatment to improve its cost-effectiveness. Although surface modification using nano zero-valent iron (*n*ZVI) may be effective to enhance its performance, the production cost of CF for water treatment is high.

To reflect its novelty, this study investigated the technical feasibility of iron-coated carbon fiber synthesized via in situ iron reduction for the adsorption of As(III) from an aqueous solution. The resulting adsorbent was characterized using Brunauer–Emmett–Teller (BET), scanning electron microscopy (SEM), X-ray diffraction (XRD), and Fourier-transform infrared (FTIR) spectroscopy. Batch experiments were carried out under optimized conditions of reaction time, dosage, As(III) initial concentration, pH, and in the presence of coexisting ions (Mg^2+^, Ca^2+^, K^+^, Cl^−^, SO_4_^2−^, HCO_3_^−^, and PO_4_^3−^). The removal of As(III) by the Fe@CF was evaluated and compared to as-received CF and those of other materials.

## 2. Materials and Methods

### 2.1. Materials

Carbon fiber cloth (30 cm × 30 cm) was provided by the Anhui Tianfu Technology (China). The material was then crushed, passed through 60-mesh screen (<0.25 mm), and thoroughly washed with ultra-pure water. The average diameter of the fibers used in this study was less than 0.25 mm. A stock solution of As(III) (1000 mg/L) was prepared from Tanmo Standard Substances Center (Changzhou, China). Each working solution was obtained by diluting the stock solution using ultrapure water (18.2 MΩ cm^−1^). Other reagents were obtained from Adamas (Shanghai, China) and used without further purification.

### 2.2. Synthesis of Iron-Coated Carbon Fiber Composite

In a typical in situ reduction method [23,24], 3.78 g of carbon fiber was mixed with 27 mM of FeCl_2_·4H_2_O solution (200 mL). Afterward, 54 mM of KBH_4_ (200 mL) was added dropwise to the mixture, which reduced Fe^2+^ to Fe^0^ (Equation (1)) [25]. After 1 h of stirring, the resultant solid was filtered and washed repeatedly. In this process, the Cl^−^ and K^+^, which did not participate in the reaction, were removed. After the complete synthesis of the composite, the resulting material was vacuum-dried at 105 °C overnight and marked as Fe@CF.

(1) $$2Fe^{2+}+BH_{4}^{-}+2H_{2}O\to 2Fe^{0}+BO_{2}{}^{-}+2H_{2}+4H^{+}$$

### 2.3. Characterizations of CF and Fe@CF

The specific surface area and porous properties of the materials used in this study were determined on an ASAP2460 instrument (Micromeritics, Norcross, GA, USA) using BET N_2_ adsorption-desorption method. Fourier-transform infrared (FTIR) spectra were collected on a Nexus 670 (Nicole, Ramsey, MN, USA) in the range of 4000–400 cm^−1^. X-ray diffraction (XRD) measurement was carried out on an X’Pert Pro MPD X-ray diffractometer (Nalytical, Amsterdam, The Netherlands) using CuKα radiation at 40 kV, 30 mA at 5°/min. Scanning electron microscope tests (TESCAN MIRA LMS, Brno, Czech Republic) along with an Xplore 30 EDS detector were recorded to detect the surface morphology and composition of the composite.

### 2.4. Batch Adsorption Studies

The effects of reaction time (10~180 min), Fe@CF dose (0.5~3 g/L), initial concentration of As(III) (0.5~2 mg/L), pH (3.5~9.5), and coexisting ions with an initial concentration of 0.1 M (cations: Na^+^, Mg^2+^, and Ca^2+^; anions: Cl^−^, HCO_3_^−^, SO_4_^2−^, and PO_4_^3−^) were examined to determine the optimum conditions of As(III) removal by Fe@CF. The batch experiments were conducted at ambient temperature. After the reaction was complete, an aliquot of sample solution was collected and filtered through a 0.45 μm microporous membrane by using 5 mL of plastic injector. As the size of the Fe@CF composite was less than 0.25 mm, the spent composite can be recovered from the contaminated water through 0.45 μm micro-porous membrane. The saturated adsorbent could be regenerated and reused for subsequent treatment [26].

### 2.5. Analysis Method

The remaining concentration of As(III) after treatment was measured by hydride generation atomic fluorescence spectrometry (HG-AFS, SA-20, Titian, China). The As(III) was converted to AsH_3_ by 2% (*w*/*v*) KBH_4_ in 0.5% (*w*/*v*) NaOH solution (Equation (2)). Citric acid (0.1 M) was used as carrier solution, while 0.4 M sodium citrate buffer (pH 4.5) was used as the working solution.

(2) $$As^{3+}+4BH_{4}^{-}+H^{+}+8H_{2}O\to AsH_{3}↑+4HBO_{2}↑+13H_{2}↑$$

The As(III) removal (η(%)) was calculated based on the standard method [27] as follows:

(3) $$\eta (\%)=(1-\frac{C_{e}}{C_{0}})\times 100$$

where: C_0_ and C_e_ are the initial and equilibrium concentration of As(III), respectively.

### 2.6. Statistical Tests

The adsorption experiments were conducted in triplicate. Their means were presented with their coefficient variations of less than 5%. Statistical analysis was conducted using SPSS 25.0 Windows version. Differences were statistically significant when *p* ≤ 0.05.

## 3. Results and Discussion

### 3.1. Physico-Chemical Properties of Fe@CF

**Figure 1:**
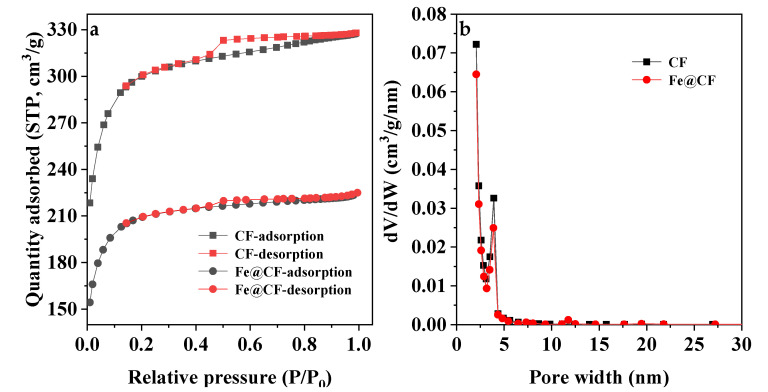
N_2_ adsorption–desorption isotherm curves (**a**) and pore size distribution curves (**b**) of CF and Fe@CF.

The N_2_ adsorption–desorption isotherms curves and pore size distribution of the CF and Fe@CF composite are presented in Figure 1. Compared to those of the as-received CF (776.1 m^2^/g and 0.37 cm^3^/g), the BET surface area and pore volume of the Fe@CF were slightly smaller (717.8 m^2^/g and 0.35 cm^3^/g), respectively (Figure 1a). The reduced BET surface area and pore volume of the Fe@CF were ascribed to the coating of the Fe nanoparticles. In addition, the adsorption–desorption curves of both the CF and Fe@CF reached 0.2 of relative pressure and the average pore size was 2.9 nm (Figure 1b), indicating that both the materials had a highly ordered mesoporous structure. No pore blockage occurred on the surface of Fe@CF by Fe^0^. The findings were consistent with the results reported earlier by Qu et al. [25].

**Figure 2:**
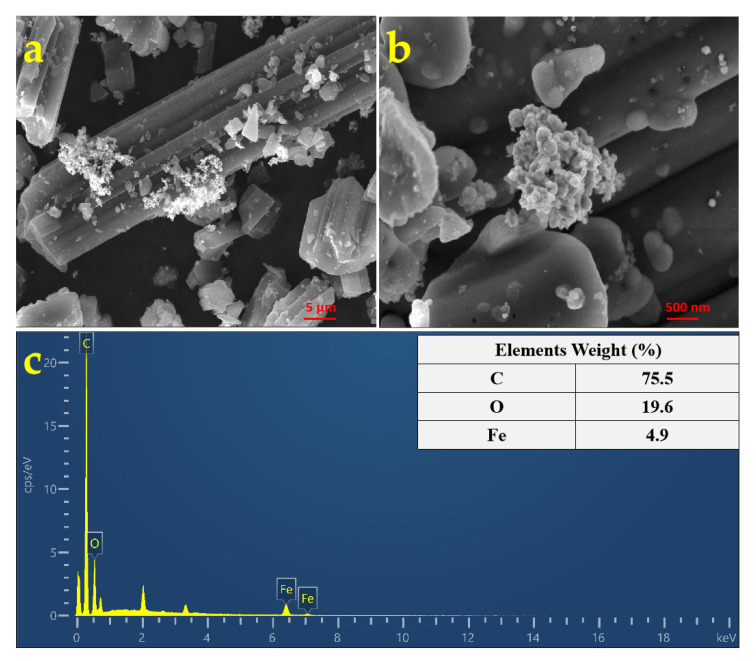
SEM images (**a**,**b**) and the EDS spectrum (**c**) of Fe@CF.

SEM images were used to understand the morphological characteristics of the Fe@CF composite. The synthesized Fe nanoparticles coated on the columnar-shaped CF showed a globular-like morphology with an average particle size of 116 nm and formed an aggregated structure due to their intrinsic magnetic property. This characteristic represents the magnetic attractive force between particles that increases with the sixth power of particle/agglomerate radius [28], which might result in decreasing the adsorption of the target pollutant. A higher Fe^0^ content in the composite was attributed to their agglomeration (Figure 2a,b). The EDS spectrum of the selected area, presented in Figure 2c, shows that the elements of C, O, and Fe existed in the Fe@CF composite. The weak peaks of the Fe were observed and its weight fraction in the Fe@CF composite was 4.9% (Figure 2c). The results indicate that the Fe nanoparticles were successfully anchored on the surface of the CF.

**Figure 3:**
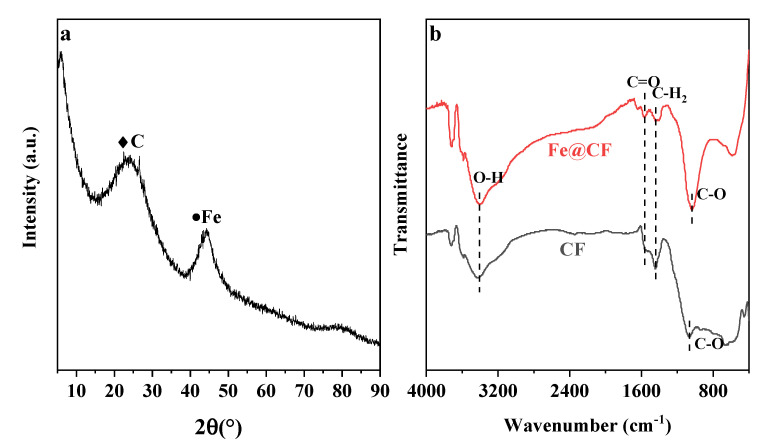
XRD pattern of Fe@CF (**a**); FTIR spectra of CF and Fe@CF (**b**).

The XRD pattern of Fe@CF is presented in Figure 3a. The diffraction peak at 22.5° was ascribed to amorphous carbon, while the peak at 44.9° was indexed to the 110-plane reflection of the metallic α-Fe (Fe^0^) (JCPDS NO. 06-0696) [15]. This indicated that the CF was coated by nZVI, which was consistent with the results of the EDX. No other diffraction peaks were observed, suggesting that nZVI was the main species coated onto the CF.

FTIR studies in the region of 4000~400 cm^−1^ were carried out to identify changes in the oxygen-containing groups on the surface of CF before and after surface modification with nZVI. Figure 3b shows that the broad band that appeared at 3410 cm^−1^ was assigned to the -OH stretching vibrations. Weak bands around 1560 cm^−1^ corresponded to the vibration of C=O [29], while the broad and intense band at 1040 cm^−1^ could be attributed to the C-O vibration [22]. The results suggested that the oxygen-containing groups on the CF had no obvious changes after being coated with nZVI. The broad peak below 800 cm^−1^ was responsible for the Si-O-Si stretching. The band of Si-O-Si at Fe@CF decreased, suggesting a reaction between Si-O-Si and nZVI [30]. Numerous studies reported that the stretching of the Si-O-Si variation resulted from the entrapment of the Fe atom by SiO_2_. The bond of the Si-O-Fe was attributed to the compacted coating of silica on the Fe nanoparticles [31].

### 3.2. Effect of Reaction Time on As(III) Removal

**Figure 4:**
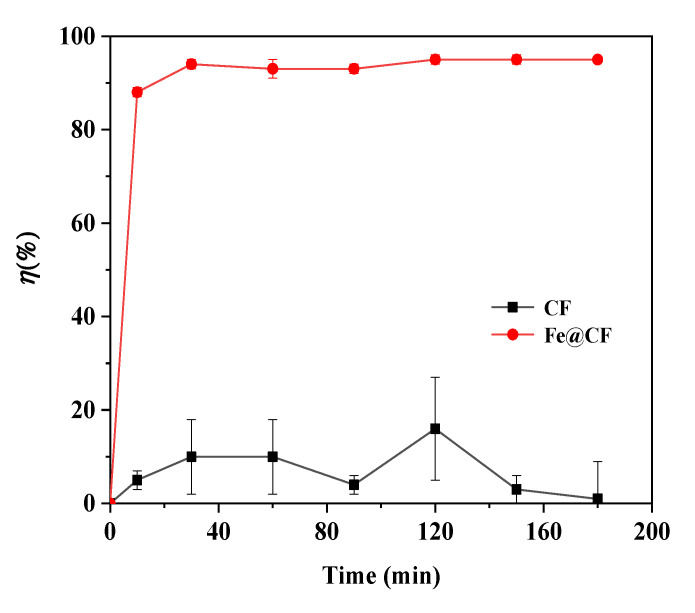
Effect of contact time on As(III) adsorption by Fe@CF. (Experimental conditions: 1 g/L of dosage, 1 mg/L of As(III), pH 3.5, 150 rpm, 25 °C).

The adsorption of As(III) by CF and Fe@CF was studied as a function of contact time at the same concentration of 1 mg/L. Figure 4 shows that the adsorption of As(III) by CF remained unchanged for 3 h of reaction time. In contrast, the adsorption of As(III) by the Fe@CF composite increased sharply with the longer reaction time. About 94% of As(III) (C_0_ = 1 mg/L) was adsorbed within 30 min. The high rate of As(III) uptake at the initial stage of adsorption was ascribed to the availability of active sites on the composite. The difference in the adsorbate concentration between the solution and the adsorbent surface provided a driving force for mass transfer [31]. The adsorption of As(III) by the composite achieved an equilibrium after 30 min, implying that the active sites were gradually occupied by the adsorbate [32]. This indicated that the Fe@CF had not only larger adsorption sites, but also a short equilibrium time. Overall, a short reaction time was essential to minimize treatment costs [33]. Therefore, 30 min of contact time was adopted for subsequent studies.

### 3.3. Effect of Dose on As(III) Removal

**Figure 5:**
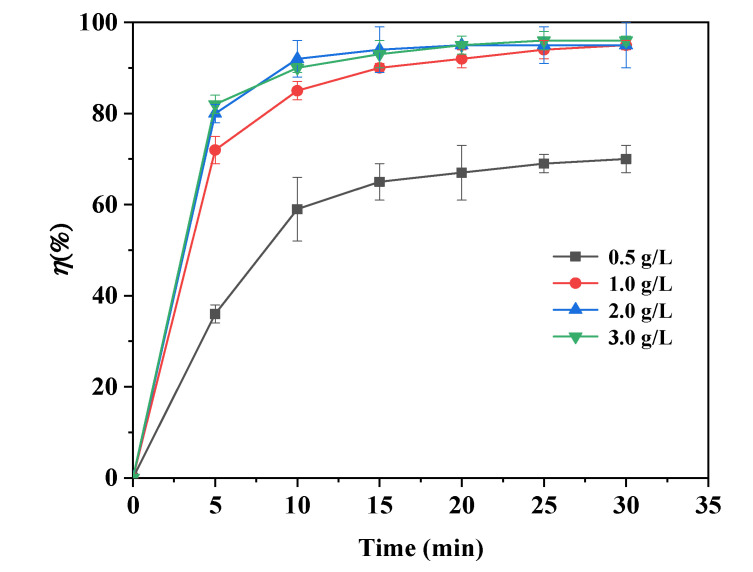
Effect of dosage on As(III) adsorption by Fe@CF as a function of reaction time. (Experimental conditions: 0.5~3.0 g/L of dosage, 1 mg/L of As(III), pH 3.5, 150 rpm, 25 °C).

The effects of dose on the adsorption of As(III) by Fe@CF were tested by varying the dose from 0.5 to 3.0 g/L. Figure 5 shows that the adsorption of As(III) by the Fe@CF was enhanced with an increasing dose. The As(III) removal efficiencies by the Fe@CF were 70, 95, 95, and 96% with a varying dose of 0.5, 1.0, 2.0, and 3.0 g/L, respectively. In general, an increasing dose would provide additional sites for adsorbing the target pollutant [34,35]. Optimum removal of As by the Fe@CF was achieved at 1.0 g/L of dose.

With the varying dose from 1.0–3.0 g/L, there was no significant increase in the As(III) adsorption. A large amount of adsorbent led to particle aggregation, which resulted in an overall reduction in the adsorbent–adsorbate interactions [36,37].

### 3.4. Effect of Initial Concentration of As(III) on Its Removal

**Figure 6:**
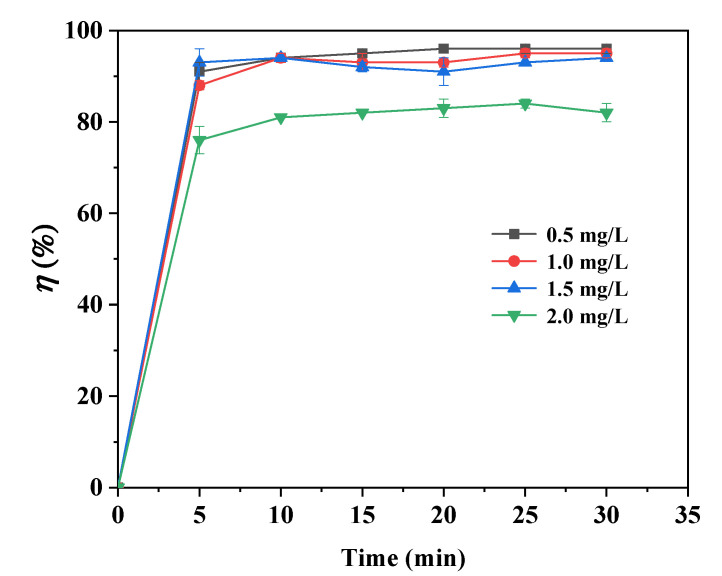
Effect of initial As(III) concentrations on its adsorption by Fe@CF. (Experimental conditions: 1 g/L of dosage, 0.5~2.0 mg/L of As(III), pH 3.5, 150 rpm, 25 °C).

The effects of initial As(III) concentrations on its adsorption by the Fe@CF were studied in the range of 0.5~2.0 mg/L while keeping other parameters constant (30 min of reaction time and 1 g/L of dosage). As depicted in Figure 6, the As(III) adsorption was over 95% at 1.5 mg/L of As(III) concentrations after 30 min of reaction and decreased to 84% when the As(III) concentration increased to 2.0 mg/L (*p* > 0.05; ANOVA test). A similar trend was also reported for the adsorption of As(V) on nZVI-supported by activated carbon [38]. The decrease in As(III) adsorption was attributed to less availability of the active sites for a certain amount of dose [39]. The active sites available for adsorption were substantial at low As(III) concentrations. However, as the active sites on the adsorbent became saturated at higher As(III) concentrations, the adsorbent became increasingly exhausted [40].

### 3.5. Effect of pH on As(III) Removal by Fe@CF

Optimum pH affects the adsorption behavior by controlling the surface charge of the adsorbent and the chemical speciation of adsorbate [8,41]. For instance, As(III) is stable at pH 0~9 as neutral H_3_AsO_3_, while H_2_AsO_3_^−^, HAsO_3_^2−^, and AsO_3_^3−^ exist as stable species at pH ranging from 10~14 [42]. Therefore, the adsorption of As(III) by the Fe@CF was studied at varying pHs from 3.5 to 9.5.

**Figure 7:**
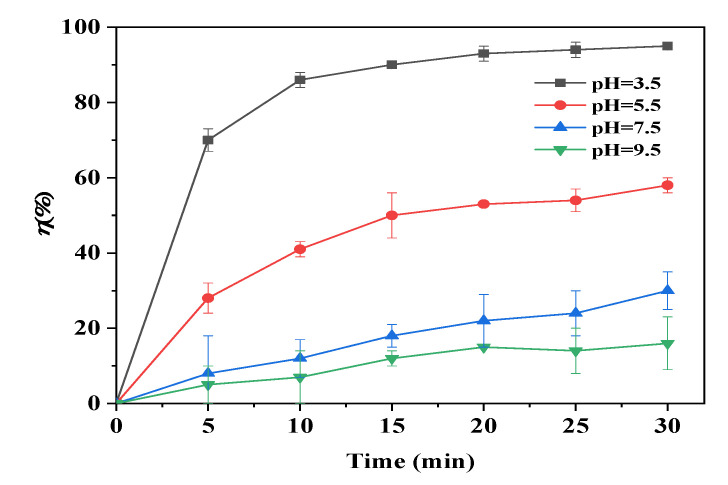
Effect of pH on As(III) adsorption by Fe@CF. (Experimental conditions: 1 g/L of dosage, 1 mg/L of As(III), pH 3.5~9.5, 150 rpm, 25 °C).

Figure 7 shows that the maximum adsorption of As(III) by the Fe@CF was 95%, suggesting that low pH was beneficial to the adsorption of As(III) by the Fe@CF. It should be noted that the initial pH of 1 mg/L of As(III) solution was 3.5. Therefore, the optimum As(III) adsorption at pH 3.5 did not require pH adjustment.

H_3_AsO_3_ formation might reduce the interaction between As(III) with Fe@CF because the As(III) ions preferred to form a compound rather than to be adsorbed on the surface of Fe@CF. In an acidic environment, the high As(III) removal was attributed to H-bonding and the electrostatic attraction between the As speciation (H_3_AsO_3_) and the positively charged Fe@CF [43]. As the pH of the solution increased from 3.5 to 9.5, the H_3_AsO_3_ in the solution was gradually converted to H_2_AsO_3_^−^, while protons were released from the C-OH group on the Fe@CF [44]. The negatively charged Fe@CF created charge repulsion with the As(III) species, reducing the adsorption of As(III).

### 3.6. Effect of Coexisting Ions on As(III) Removal by Fe@CF

**Figure 8:**
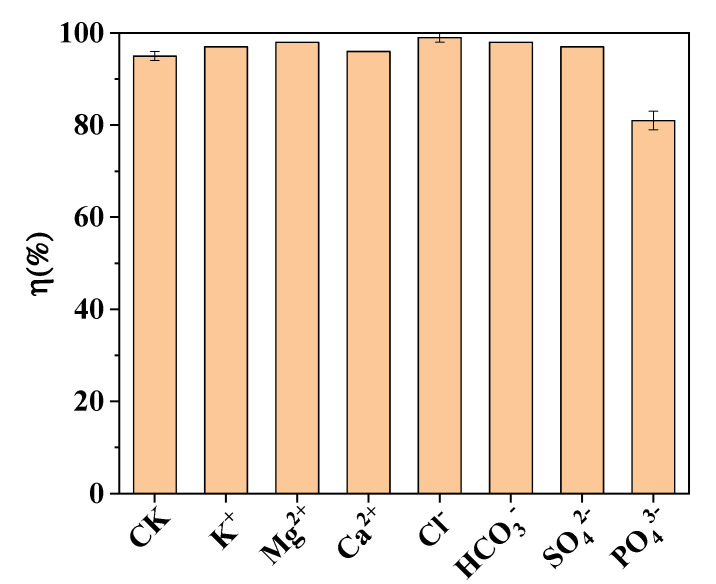
Effect of coexisting ions on As(III) removal by Fe@CF. (Experimental conditions: 1 g/L of dose, 1 mg/L of As(III), 0.1 M of coexisting ions, pH 3.5, 150 rpm, 25 °C; CK represents control).

Figure 8 shows the effects of coexisting ions (Na^+^, Ca^2+^, Mg^2+^, Cl^−^, HCO_3_^−^, SO_4_^2−^, and PO_4_^3−^) on As(III) removal by the Fe@CF at pH 3.5. Initial As(III) concentration was 1 mg/L and the concentration of competitive ions was 0.1 M. As depicted in Figure 8, Na^+^, Ca^2+^, and Mg^2+^ had negligible effects on As(III) adsorption, implying that the cations were hardly adsorbed by the protonated composite because of the electrostatic repulsion. Cl^−^, HCO_3_^−^, and SO_4_^2−^ did not interfere in the As(III) removal by the Fe@CF, while the counterproductive impact on As(III) adsorption took place due to the presence of PO_4_^3−^ (0.1 M) under the same conditions.

As the As(III) was removed from the wastewater solution as an anion, the selectivity trend depended on the Hofmeister series. Due to their hydrophobicity, certain materials prefer lowly hydrated anions to highly hydrated ones due to their charge numbers. This implies that the higher valences are more predominant than the lower valence ions based on the Hofmeister series [45]. Another explanation is that PO_4_^3−^ is a competitor with As(III) species for adsorption sites on the Fe@CF [46,47,48], due to its similar tetrahedral structure to that of arsenic [49]. This was also supported by Wei et al. [50], who also reported that Ca^2+^, Mg^2+^, and SO_4_^2−^ ions did not substantially affect As(III) adsorption due to their low affinity with Fe, as compared to the As(V).

### 3.7. As(III) Adsorption Mechanisms on Fe@CF

Based on the results above, the Fe@CF composite showed an excellent As(III) adsorption with a maximum capacity of 1.6 mg/g, which was due to the presence of nZVI on the CF surface (Figure 4). Previous studies found that the nZVI served as the adsorption site for As(III) in aqueous media, while the CF functioned as a carrier to distribute and stabilize the nZVI. As(III) removal by nZVI involved As(III) oxyanions that formed inner-sphere surface complexation with hydroxyl groups of Fe oxides [51].

**Figure 9:**
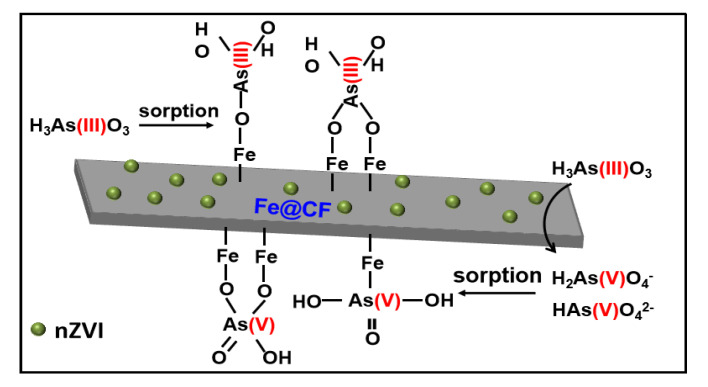
Proposed adsorption mechanisms of As(III) by Fe@CF.

Liu et al. [52] clarified the roles of carbonaceous supports in enhancing As(III) removal by nZVI. The electron-accepting capacity of the carbonaceous supports was associated with As(III) oxidation and its removal by nZVI [53]. The inner-sphere surface complexation of As(III) or As(V) with Fe oxides represents the main mechanisms of As(III) removal (Figure 9).

### 3.8. Comparison of As(III) Removal between Fe@CF and Other Adsorbents in Batch Studies

The removal performance of As(III) from aqueous solutions using various adsorbents is presented in Table 1. Compared to the other adsorbents, the Fe@CF composite was an outstanding adsorbent that could be exploited for As(III) removal. It had the highest As(III) adsorption capacity. The findings reveal favorable implications for environmental engineers as Fe@CF can be utilized effectively to facilitate effective remediation in the aquatic environment. However, the treated effluents could not meet the strict discharge standard limit of ≤10 μg/L set by the WHO. A longer reaction time was required to complete the removal of the remaining As(III) in the effluents, increasing the operational cost of wastewater treatment.

## 4. Conclusions

This study has demonstrated the feasibility of the iron-coated CF composite for an effective As(III) adsorption from aqueous media. The SEM and XRD analyses confirmed that the CF was successfully anchored by Fe(0) nanoparticles during in situ fabrication. During treatment, 95% of As(III) was adsorbed at optimized conditions (30 min of reaction time, 1 g/L of dose, 1 mg/L of As(III) concentration, and pH 3.5). With the increasing regeneration tests, the adsorptive ability of the Fe@CF composites might gradually decline. Since treated effluents could not meet the strict discharge standard limit of ≤10 μg/L set by the WHO’s regulation, a longer reaction time was necessary to complete the removal of As(III) in the wastewater. As compared to the other adsorbents reported previously, the Fe@CF composite had the highest As(III) adsorption. Overall, the findings suggested that the Fe@CF was promising to facilitate an effective As(III) remediation from contaminated water.

## Figures and Tables

**Table 1:** Comparison of the As(III) adsorption of various materials.

Adsorbent	Maximum Adsorption (%)	Optimum Conditions	Reference
Fe@CF	95	1 g/L of dosage, 1 mg/L of As(III), 30 min, pH 3.5	This study
C-mVMT	94	20 g/L of dosage, 10 mg/L of As(III), pH 5	[36]
Fe_3_O_4_/SS-BC	93	2 g/L of dosage, 0.5 mg/L of As(III), pH 5	[54]
Fe@CTS ENM	85	0.3 g/L of dosage, 0.1 mg/L As(III), 24 h, pH = 3.3~8.0	[55]
Fe_3_O_4_/AC	70	1.8 g/L of dosage, 5 mg/L of As(III), 60 min, pH 8.0	[56]

## Data Availability

Data presented in this study are available in the article.
